# Notifiable Disease and Mortality Tables for Weeks 39–41 Now Online

**Published:** 2013-10-25

The Notifiable Disease and Mortality Tables for surveillance weeks 39, 40, and 41 have now been posted on the *MMWR* website, along with current week 42 data and the October 25, 2013, issue. The data include quarterly Table IV data regarding tuberculosis. Posting of the notifiable disease and mortality data for the 3-week period was delayed because of the lapse in government funding.

